# Supplementary material for: Multi-Class Cancer Subtyping in Salivary Gland Carcinomas with MALDI Imaging and Deep Learning
Source: Cancers (Basel). 2022 Sep 5;14(17):4342. doi: 10.3390/cancers14174342 (PMC9454426; doi:10.3390/cancers14174342)
Supplement: Supplementary file 1 [file cancers-14-04342-s001.zip › cancers-1843380-supplementary.pdf]

Tumor Subtype	T	N	sex	Age_diag
Acin	4a	2	w	64
Acin	4a	2	w	64
Acin	3	0	m	46
Acin	2	0	m	42
Acin	1	0	w	64
AdCy	2	0	w	66
AdCy	1	0	w	38
AdCy	1	0	m	40
AdCy	3	0	m	71
AdCy	4a	2	w	58
AdCy	1	0	m	52
AdCy	NA	0	w	75
ANOS	3	3	m	NA
ANOS	3	0	w	61
MuEp	3	2	m	77
MuEp	2	0	m	34
MuEp	2	0	w	17
SaDu	3	2	w	61
Sec	3	0	w	60

Table S1: Clinicopathological data of SGC patient cohort. AdCy: adenoid cystic carcinoma; MuEp: mucoepidermoid carcinoma; SaDu: salivary duct carcinoma; Acin: acinic cell carcinoma; Sec: secretory carcinoma, ANOS: adenocarcinoma not-otherwise-specified. T and N according to the UICC TNM cancer staging system.

Acin	AdCy	Anos	MuEp	SaDu	Sec
1724.39	892.5231	1362.271	1893.147	1816.258	1022.111
833.0767	1952.387	645.0326	1589.264	1818.251	1023.114
1724.384	1725.393	833.0832	1725.393	1854.198	1251.116
1220.604	1574.293	1220.604	1590.254	1386.223	1521.304
1893.147	1401.207	644.0298	1173.218	1817.241	1022.117
1362.271	1213.176	1174.227	852.4349	1817.267	1627.204
1401.214	985.1735	1401.22	1590.261	1817.254	1022.13
1725.393	1173.218	1537.349	644.0298	985.1735	1023.12
1537.349	1401.214	833.0767	606.0832	1817.248	1023.126
1401.22	1401.22	1173.218	985.1735	1627.204	606.0832

Table S2: Most significant masses (m/z) for each tumor subtype according to 3.3. sorted in ascending order. AdCy: adenoid cystic carcinoma; MuEp: mucoepidermoid carcinoma; SaDu: salivary duct carcinoma; Acin: acinic cell carcinoma; Sec: secretory carcinoma, ANOS: adenocarcinoma not-otherwise-specified.

Figure S1: Interactive dense map clustering viewable in web browser. Mouse wheel zoom can be enabled via “Wheel Zoom” button. Each point represents one mass spectrum and on hover displays the TMA core of origin. SGC subtype 1: acinic cell carcinoma; SGC subtype 2: adenoid cystic carcinoma; SGC subtype 3: adenocarcinoma not-otherwise-specified; SGC subtype 4: mucoepidermoid carcinoma; SGC subtype 5: salivary duct carcinoma; SGC subtype 6: secretory carcinoma. Created using Bokeh, D. T. "Bokeh: Python library for interactive visualization." (2018).
