# Supplementary material for: Multi-Class Cancer Subtyping in Salivary Gland Carcinomas with MALDI Imaging and Deep Learning
Source: Cancers (Basel). 2022 Sep 5;14(17):4342. doi: 10.3390/cancers14174342 (PMC9454426; doi:10.3390/cancers14174342)
Supplement: Supplementary file 1 [file cancers-14-04342-s001.zip › cancers-1843380-Figure S1.html]

Static HTML file
